# Psychometric analysis of the Swedish version of the General Medical Council's multi source feedback questionnaires

**DOI:** 10.5116/ijme.5948.0bb6

**Published:** 2017-07-10

**Authors:** Jan-Eric Olsson, Fan Yang Wallentin, Eva Toth-Pal, Solvig Ekblad, Bo Christer Bertilson

**Affiliations:** 1 Academic Primary Healthcare Centre, Huddinge, Sweden; 2 Department of Statistics, University of Uppsala, Sweden; 3 Division of Family Medicine, Department of Neurobiology, Care Sciences and Society, Karolinska Institutet, Stockholm, Sweden

**Keywords:** Multisource feedback questionnaires, physicians, assessment, factor analysis, validation

## Abstract

**Objectives:**

To determine the internal consistency and the underlying components of our translated and adapted Swedish version of the General Medical Council's multisource feedback questionnaires (GMC questionnaires) for physicians and to confirm which aspects of good medical practice the latent variable structure reflected.

**Methods:**

From October 2015 to March 2016, residents in family medicine in Sweden were invited to participate in the study and to use the Swedish version to perform self-evaluations and acquire feedback from both their patients and colleagues. The validation focused on internal consistency and construct validity. Main outcome measures were Cronbach’s alpha coefficients, Principal Component Analysis, and Confirmatory Factor Analysis indices.

**Results:**

A total of 752 completed questionnaires from patients, colleagues, and residents were analysed. Of these, 213 comprised resident self-evaluations, 336 were feedback from residents’ patients, and 203 were feedback from residents’ colleagues. Cronbach’s alpha coefficients of the scores were 0.88 from patients, 0.93 from colleagues, and 0.84 in the self-evaluations. The Confirmatory Factor Analysis validated two models that fit the data reasonably well and reflected important aspects of good medical practice. The first model had two latent factors for patient-related items concerning empathy and consultation management, and the second model had five latent factors for colleague-related items, including knowledge and skills, attitude and approach, reflection and development, teaching, and trust.

**Conclusions:**

The current Swedish version seems to be a reliable and valid tool for formative assessment for resident physicians and their supervisors. This needs to be verified in larger samples.

## Introduction

Measurable criteria for good medical practice are needed to assess competence and to give feedback to physicians in their development. Being a good physician requires relevant clinical knowledge, adherence to common guidelines, and commitment to follow basic ethical tenets with the patient's safety and health as the main goal.^1^ Unfortunately, there is some evidence that physicians have limited ability to assess their own competence and compare it with external observations.^2^ Feedback and assessment from colleagues and patients promotes learning and appropriate development,^3^ and by using validated questionnaires to collect these perspectives, such feedback can contribute to the Work Place-Based Assessments (WPBA) increasingly used in many countries.^4^ Multisource feedback (MSF) refers to a WPBA tool with high reliability, validity, and feasibility that is often used in English-speaking countries in order to assess physicians’ clinical competence.^5^ MSF is a method in which colleagues, co-workers, and patients make overall assessments and give feedback to physicians in their clinical practice, and it is well proven to assess interpersonal communication, professionalism, and teamwork behaviors.^5^ However, the method is not without its disadvantages. “MSF is not a replacement for auditing when clinical outcomes need to be assessed” according to Lockyer.^6^ The ability of MSF to identify poor performance due to leniency bias from chosen raters has been raised as a potential weakness, as has the potential impact of combining scores from patients with colleague feedback.^7^

One internationally known and widely used MSF tool is “The General Medical Council Multi-Source Feedback Questionnaires” (GMC MSF Questionnaires) developed in the UK.^8^ In the following text we will refer to the questionnaires as the GMC questionnaires. The questionnaires are based on the GMC's guidance on good medical practice for physicians.^9^ 

The GMC defines four domains of good medical practice that are reflected in the items of the GMC questionnaires. The first domain includes medical knowledge, skills, and performance.  Domain two is about safety and quality, and domain three is about communication, partnership, and teamwork. The fourth domain concerns maintaining the trust of patients and colleagues by acting with honesty and integrity. The GMC questionnaires, which are used for revalidation of physicians, were developed from the English-language General Practice Assessment Survey (GPAS) 2000.^10^ A comprehensive validation of the GMC questionnaires was done in the UK in 2008–2012 on 1,057 physicians who received feedback from 17,012 colleagues and 30,333 patients,^8^^,^^11^ and this validation included analysis of principal components, internal consistency, convergent validity, generalizability, feasibility, and acceptability. The Cronbach’s alphas in the UK study were 0.87 for patients and 0.94 for colleagues. A vast majority of index physicians were assessed in the two highest scores by both patients and colleagues. The response option of ‘does not apply’ varied from 1 to 28% across individual items in the colleague and patient questionnaires. Two ‘patient components’ and three ‘colleague components’ had emerged from the UK Principal component analysis (PCA).^12^

Confirmatory factor analysis (CFA) has, to our knowledge, not been performed to analyse which latent dimensions in the GMC questionnaires are reflected by the scores from patients, colleagues and including self-evaluating residents. However, construct validity in the GMC questionnaires might be supported by CFA if the scores from residents and patients reflect the same patient-related latent dimensions of good medical practice and if the scores from residents and colleagues reflect the same colleague-related latent dimensions of good medical practice.

In Sweden, residents perform annual self-evaluations, and individual external assessments are carried out by senior colleagues once during their resident period. The number of registered residents in family medicine in Sweden was just above 2,000 in 2013, and one quarter of them were registered in Stockholm County Council. Each resident is assigned a personal tutor for support in clinical and professional issues. However, validated Swedish MSF instruments assessing good medical practice have to date been lacking in Sweden.

The purpose of creating a Swedish version of the GMC questionnaires was to provide a scientifically tested MSF tool for feedback and competence development for resident physicians and their supervisors during residency. This tool could serve as a complement to existing assessment methods and add new pedagogical opportunities for supervision of resident physicians.

In an earlier study we translated and adapted the GMC questionnaires to a Swedish context (manuscript in preparation). A translation and back-translation of the GMC questionnaires was performed by professional translators. After a second revision by a panel of experts, we conducted semi-structured interviews with a total of 103 residents, patients, and colleagues in order to collect their views on the questionnaires in general and their interpretation of and comments to the translated text. The results were incorporated in the adapted Swedish version.

In this article we report the results from our psychometric analysis of the adapted Swedish version. Our aim was to assess if the Swedish version met adequate scientific requirements for internal consistency and construct validity.

## Methods

### The Swedish questionnaires

**Figure 1:**
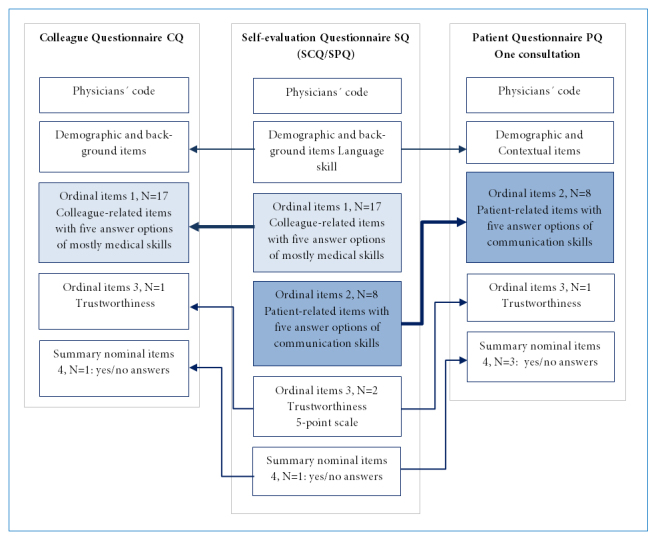
The structure of the adapted Swedish version of the three GMC questionnaires. The self-evaluation questionnaire SQ consists of two parts, colleague-related items (SCQ) and patient-related items (SPQ). Corresponding items in the colleague questionnaire CQ and the patient questionnaire PQ are marked with arrows.

The Swedish version of the GMC questionnaires consists of three components with partly similar content: a patient questionnaire (PQ), a colleague questionnaire (CQ), and a questionnaire for self-evaluation (SQ), with 22, 29, and 34 items respectively, including demographic and contextual items. The structure of the common items in the three questionnaires is explained in Figure 1. The SQ itself is divided into two parts: a part with patient-related items (SPQ) and one with colleague-related items (SCQ). Ten patient-related items are common in the PQ and SPQ, and these items concern consultation skills, patient acceptance, and aspects of the physician’s trustworthiness. The PQ is intended for the patient to answer directly after a consultation. The common parts in the CQ and SCQ include 22 colleague-related items that assess physicians' clinical, communication, organizational, and educational skills and aspects of their trustworthiness. All questionnaires can be answered in both paper and electronic format. Physicians using the GMC questionnaires are able to compare their self-evaluation with the answers from patients and colleagues.

### Study design and participants

Data collection was conducted from October 2015 to March 2016. Participating physicians were recruited from residents in family medicine in various parts of Sweden by e-mail or at meetings. Bulk e-mail invitations were initially sent to several hundred randomly selected residents in family medicine with an invitation to participate in the study. The response rate was less than 10%. We, therefore, switched strategies and offered residents who attended meetings to perform a self-evaluation during the meeting and then to proceed with collecting feedback from patients and colleagues. The response rate for the SQ increased to 85%–92%. We planned to reach at least 200 answers for each of the three questionnaires to get adequate sample size. We were, however, aware that only a minority of self-evaluating residents would go on to get assessment from external assessors.

Residents who registered for the study received the web-link addresses for the online versions of the CQ and PQ and allocated a personal code known only to the administrative secretary of the study. Patients were invited by a receptionist at the clinic where the resident worked to give anonymous feedback either by paper questionnaires marked with the physician's code or by coded web surveys. Residents were unaware of when and which patients were invited to give feedback. Participating residents could then email the code and the web link to a number of colleagues who were chosen by themselves to give anonymous feedback. The residents were asked to gather at least 34 patient surveys, and 12 colleague surveys (in line with UK recommendations) or as many as possible.^12^

### Data analysis

Internet based software was used for distribution of online questionnaires and for collection, and processing of the responses. Paleontological Statistics (PAST version 3.15)^13^ was used for descriptive statistics. IBM SPSS (version 22)^14^ and LISREL software (version 9.2)^15^ was used for statistical analysis. SPSS was used for the calculation of Cronbach’s alpha and principal components analysis (PCA).  PCA with maximum likelihood extraction (based on Eigenvalue >1) and oblimin rotation with Kaiser Normalization preceded by The Kaiser-Meyer-Olkin Measure of  Sampling Adequacy (KMO) and Bartlett’s Test, were carried out to get an indication of the underlying components of the 5-point scale items (Figure 1). KMO was used as a measure of the proportion of common variance among variables (0.8–1 indicates adequate sampling).^16^ Bartlett’s Test was used to verify equal variances across samples. (p<0.001 verifies equal variance).^17^

To confirm which aspects of good medical practice the latent variable structure reflected, a CFA was performed using the maximum likelihood estimation method in the LISREL software package. In order to overcome the problem of missing values in LISREL, we used multiple imputations, which is a statistical technique for analyzing incomplete datasets.^18^  Listwise deletion was used for missing values for all calculations in SPSS except for Mann-Whitney’s test where test-by-test exclusion was used. Corrected item-total correlations exceeding 0.30 were regarded as acceptable.^19^ Significance in the χ^2^, Kruskal-Wallis and Mann-Whitney’s tests were defined as p < 0.05.

The goodness-of-fit statistical measures were applied to test how well the defined model fit the data. Each fit class in the goodness-of-fit analysis provides different information about the model fit, and at least one index from each fit class was analysed to provide information about the fit of the CFA solution.^20^ To evaluate the overall model fit, the following fit indices were applied: A chi-square (χ^2^) test was calculated to test the fit of the model. Relative χ^2^ (χ^2^/degrees of freedom (df)) was used, and acceptable threshold levels were 2:1–3:1.^21^ The standardized root mean square residual (SRMR) is the square root of the difference between the residuals of the sample covariance and the hypothesized covariance model. Values for the SRMR range from 0 (indicating perfect fit) to 1.0. SRMR values <0.05 indicate well-fitting models, and values as high as 0.08 are deemed acceptable.^22^ The root mean square error of approximation (RMSEA) is a measurement of the model fit. RMSEA ≤ 0.05 indicates a close fit, and RMSEA > 0.05 and < 0.08 indicates an acceptable fit of the model to the data.^21^ Goodness-of-fit

 index (GFI) values range between 0 and 1, with larger values indicating better fit. A GFI value ≥0.90 is considered to indicate acceptable model fit.^23^ Factor loadings exceeding 0.30 were regarded as acceptable, and t-values ≥2 are considered to be significant (p ≤ 0.05).^19^^,^^24^^,^^25^

The sample size needed to test the criteria of the overall model fit for the CFA was decided upon using the rule of thumb of ten responses per question according to common scientific practice ^26^ because we did not find any comparable MSF CFA in the literature. This corresponded to approximately 100 questionnaires for the PQ and at least 200 questionnaires for the SQ and CQ.

### Demographic data of respondents 

Data from 752 respondents in the PQ, SQ, and CQ surveys were analysed, and demographic data are presented in Table 1. The participating residents worked in many different regions of Sweden. A total of 213 residents answered the self-evaluation, and of those 16 (13%) received feedback from both colleagues and patients.  In total, 50 residents (23%) received feedback from either patients (20 residents) or colleagues (30 residents). Feedback from 336 patients and from 203 colleagues was collected. The median numbers and quartiles (Q1–Q3) of surveys per resident were 19 surveys for patients (6–23) and 8 surveys for colleagues (4-9). According to the χ^2^ test, there were no statistical differences between the residents who received feedback and those who did not in terms of gender, the length of their residency, or country of graduation.

The English questionnaires are not copyrighted, and the project officer in the UK, Professor John Campbell, gave us permission to use the GMC questionnaires in Sweden on May 7, 2014. Ethical approval for the study was obtained from the Regional Ethical Review Board in Stockholm on 4^th^ December 2014.

## Results

### Item analysis and internal consistency

Responses on patient-related items on a five-point scale from the PQ and the self-evaluation PSQ are shown in Table 2a  and Table 2b. Responses regarding colleague-related items in the CQ and SCQ are presented in Table 3. Responses from patients and colleagues were negatively skewed with 77% in the highest scores in the PQ. In all 17 colleague-related items, 212 residents rated themselves significantly lower (mean ranks between 124.14 -176.88) than corresponding scores from 191 colleagues (mean ranks between 228.97 -279.10) according to Kruskal-Wallis test.In all 17 tests χ^2^ were between 27.92-181.28 and p<0.000. In a subgroup analysis of our SQ data we found a significant improvement between 97 residents in the first and 93 in the second part of their residency concerning clinical decision making according to Mann-Whitney U test (U=3527, p= 0.003). The proportions of “don´t know” answers on the CQ was on average 19% for all respondent groups and ranged from 1% to 50%, with the highest proportion from other personnel concerning the “supervising colleagues” question.

The Cronbach's alpha indexes of MSF scores were 0.88 from patients, 0.93 from colleagues, and 0.84 for the self-evaluations (Tables 4 and 5). The majority of corrected-item total correlations in all questionnaires were clearly over 0.30 for all patient-related and colleague-related items except for how physicians evaluated their own respect for secrecy and how colleagues evaluated trust in residents (Tables 4 and 5).

**Table 1:** Demographic data of informants in the three Swedish questionnaires 2016, by gender, age groups, professions and background

Demographic items		Patients (PQ)		Colleagues (CQ)		Self-evaluation (SQ)		Total		Rrf^*^	
		N	%	N	%	N	%	N	%	N	%
Sample size		336	45	203	27	213	28	752	100	50	23
Gender	Male	188	56	37	18	87	41	312	42	22	44
	Female	132	39	137	68	117	55	386	51	28	56
	Other	3	1	2	1	-		5	1	-	
	Missing data	13	4	27	13	9	4	49	7		
Patients, age groups	< 15 years	23	7								
	16-20	12	4								
	21-60	178	53								
	61-80	90	27								
	> 80 years	18	5								
	Missing data	15	5								
Colleagues, age groups	20-29 years			10	5						
	30-39			39	19						
	40-49			57	28						
	50-59			61	30						
	> 59 years			30	15						
	Missing data			6	3						
Colleagues, professions	Physicians			86	42						
	Nurses			67	33						
	Secretary			14	7						
	Assistant nurse			13	6						
	Others			18	9						
	Missing data			5	3						
Residents, working region in Sweden	North region					5	2			6^**^	12
	Central region					163	76			38	76
	South region					50	20			6	12
	Missing data					2	1				
Residents, country of graduation	In Sweden					123	58			33	66
	Within EU					48	23			11	22
	Outside EU					40	19			6	12
	Missing data					2	1				

^*^Residents who received feedback (Rrf) in at least one of CQ or PQ

^**^Participated in both CQ and PQ

### Principal Component Analysis

For the PQ, one component explained 55% of the total variance with loadings of 0.64–0.82. The KMO was 0.88 and Bartlett’s Test was significant. In the SPQ part, one component with loadings of 0.76–0.84 concerning the physician’s ability to solve patients’ problems explained 48% of the total variance, and the second component with negative loadings from −0.71 to −0.87 explained 15% of the variance. KMO was 0.80 and Bartlett’s Test was significant.

For the CQ, three components explained 60% of the total variance. The main component gave loadings >0.60 for 14 of the 17 colleague-related items, the second component gave high loadings for two education items, and the third component gave high loadings for two items concerning trust. The KMO was 0.92. Bartlett’s Test was significant (<0.001).

In the SCQ, five components explained 65% of the total variance. The main component with 30% of the total variance gave loadings >0.60 in 6 of the 17 colleague-related items. The second component gave high loadings in three communication items, and the third component gave high loadings in two items concerning education, while the remaining two components loaded highest in self-reflection and patient centeredness. KMO was 0.82 and Bartlett’s Test was significant.

### Confirmatory Factor Analysis

Two different factor models were defined using the PCA results and GMC’s four domains of good medical practice. Patient-related five-point scale items in the PQ and SPQ were adapted to a model with the two dimensions of empathic ability and consultation management. Results of the CFA of patient-related items are shown in Table 4. The model for the CQ and SCQ contained five factors, and the model for the PQ and SPQ contained two factors. Colleague-related five-point scale items in the CQ and SCQ (Table 5) were adapted to a model with the five dimensions of knowledge and skills, attitude and approach, teaching, reflection and development, and trust. The models did not fit the data exactly, but with some approximations the models fit the data reasonably well. All parameter estimates and all latent factor correlations for external assessors were statistically significant.

**Table 2a:** Responses from the patient questionnaire (PQ) and the self-evaluation (SPQ) concerning patient-related items*

Items back translated from Swedish	Patient questionnaire										Self-evaluation questionnaire, patient-related items									
How good was your doctor today at each of the following?	1	2	3	4	5	DN (%)	N	MD	Q1	Q3	1	2	3	4	5	DN (%)	N	MD	Q1	Q3
Give you a good reception	0	0	1	38	295	0	334	5	5	5	0	0	9	65	137	0	211	5	4	5
Making you feel secure	0	0	8	58	267	1 (3)	334	5	5	5	0	0	17	103	91	0	211	4	4	5
Listening to you	0	0	4	45	285	0	334	5	5	5	0	0	9	85	118	0	212	5	4	5
Giving you the opportunity to talk about your concerns and fears	0	0	10	59	245	20 (6)	334	5	5	5	1	0	27	98	85	1 (0.5)	212	4	4	5
Assessing your medical condition	0	4	12	82	229	6 (2)	333	5	4	5	0	1	43	134	34	0	212	4	4	5
Explaining your condition and treatment	0	2	19	62	238	13 (4)	334	5	4.8	5	0	2	48	108	53	1 (0.5)	212	4	4	5
Involving you in decisions about your treatment	0	1	7	76	237	12 (4)	333	5	4	5	0	2	46	113	50	0	211	4	4	5
Providing or arranging treatment for you	0	0	7	69	247	11 (3)	334	5	5	5	0	1	26	133	51	0	211	4	4	5

*5-point scale: 1= Insufficient, 2= Not satisfactory, 3= Satisfactory, 4= Good, 5= Very good

DN: don’t know/not applicable; MD: Median; Q1 and Q3 are answers without don’t know

**Table 2b:** Responses from the patient questionnaire (PQ) and the self-evaluation (SPQ) concerning common core performance evaluation items*

	Patient questionnaire (PQ)										Self-evaluation questionnaire (SQ)									
Response options	1	2	3	4	5	DN (%)	N	MD	Q1	Q3	1	2	3	4	5	DN	N	MD	Q1	Q3
Respecting patient confidentiality	3	0	8	51	253	9 (3)	324	5	5	5	0	0	8	77	126	0	211	5	4	5
Being honest and trustworthy	2	1	4	67	245	7 (2)	326	5	5	5	0	0	11	119	82	0	212	4	4	5
	Colleague questionnaire (CQ)																			
Response options	1*	2	3	4	5	DN (%)	N	MD	Q1	Q3										
Respecting patient confidentiality	0	0	2	23	157	17 (9)	199	5	5	5										
Being honest and trustworthy	0	0	1	22	175	2 (1)	200	5	5	5										

*5-point scale: 1= Insufficient, 2= Not satisfactory, 3= Satisfactory, 4= Good, 5= Very good

DN: don’t know/not applicable; MD: Median; Q1 and Q3 are answers without don’t know

**Table 3:** Responses to colleague-related items in the colleague questionnaire (CQ) and the self-evaluation questionnaire (SCQ)*

Items back translated from Swedish	colleague questionnaire										self-evaluation questionnaire									
Make an assessment in the following areas	1	2	3	4	5	DN (%)	N	MD	Q1	Q3	1	2	3	4	5	DN (%)	N	MD	Q1	Q3
Clinical knowledge	0	0	4	56	117	25 (12)	202	5	4	5	0	8	56	125	23	0	212	4	3	4
Diagnostic thinking	0	0	2	48	123	29 (14)	202	5	4	5	0	4	39	127	43	0	213	4	4	4
Clinical decision making	0	0	4	62	105	30 (15)	201	5	4	5	0	4	48	126	32	0	210	4	4	4
Prescribing	0	0	3	64	100	36 (18)	203	5	4	5	0	7	57	124	24	0	212	4	3	4
Treatment (Including practical procedures)	0	0	3	58	83	57 (28)	201	5	4	5	0	1	45	137	30	0	213	4	4	4
Medical record keeping	0	0	5	55	122	20 (10)	202	5	4	5	0	3	40	119	49	0	211	4	4	4
Recognising and working within own limitations	0	2	10	43	111	36 (18)	202	5	4	5	0	4	37	99	72	0	212	4	4	5
Keeping knowledge and skills up to date	0	0	4	37	126	35 (17)	202	5	5	5	0	16	74	95	27	1 (0.5)	213	4	3	4
Reviewing and reflecting on own performance	0	1	3	45	104	49 (24)	202	5	4	5	0	9	61	111	29	3 (1)	213	4	3	4
Teaching (students, trainees, others)	0	1	3	41	67	89 (44)	201	5	4	5	4	11	68	81	30	18 (8)	212	4	3	4
Supervising colleagues	0	1	2	39	58	100 (50)	200	5	4	5	2	12	72	78	19	29 (14)	212	4	3	4
Commitments to care and wellbeing of patients	0	0	3	31	152	16 (8)	202	5	5	5	0	0	16	97	97	1 (0.5)	211	4	4	5
Communication with patients and relatives	0	0	5	36	127	33 (16)	201	5	5	5	0	1	14	88	109	1 (0.5)	213	5	4	5
Working with colleagues	0	2	3	31	158	8 (4)	202	5	5	5	0	1	19	96	94	0	210	4	4	5
Effective use of time and resources	0	1	2	51	115	30 (15)	199	5	4	5	3	29	63	77	40	0	212	4	3	4
Patient-centered approach	0	0	2	36	136	28 (14)	202	5	5	5	0	1	36	104	69	1 (0.5)	211	4	4	5
Preserving the continuity of patient relationships	0	2	2	36	125	35 (18)	200	5	5	5	0	6	32	96	72	4 (2)	210	4	4	5

*5-point scale: 1= Insufficient, 2= Not satisfactory, 3= Satisfactory, 4= Good, 5= Very good

DN: don’t know/not applicable; MD: Median; Q1 and Q3 are answers without don’t know

**Table 4:** The reliability index and the confirmatory factor analysis (CFA) of patient-related items

Items back translated from Swedish	patients group N=336			self-evaluation N=213		
	factors/dimensions factor loadings (t values)		corrected item total correlation	factors/dimensions factor loadings (t values)		corrected item total correlation
How good was your doctor today at each of the following?	empathic ability	consultation management		empathic ability	consultation management	
Give you a good reception	0.62 (11.71)		0.53	0.77 (12.16)		0.55
Making you feel secure	0.80 (16.78)		0.71	0.80 (12.73)		0.64
Listening to you	0.82 (17.45)		0.71	0.75 (11.63)		0.63
Giving you the opportunity to talk about your concerns and fears	0.73 (14.88)		0.54	0.53 (7.49)		0.55
Assessing your medical condition		0.76 (15.85)	0.69		0.74 (11.07)	0.54
Explaining your condition and treatment		0.79 (16.75)	0.69		0.73 (11.39)	0.57
Involving you in decisions about your treatment		0.85 (18.75)	0.66		0.71(10.33)	0.58
Providing or arranging treatment for you		0.84 (18.19)	0.67		0.72 (11.37)	0.58
Cronbach's alpha			0.88			0.84
	Fit indices CFA for patient group:			Fit indices CFA for self-evaluation, patient part:		
	Chi-square=34.57, degrees of freedom=16 (Chi-square/df= 2.19)			Chi-square=35.57, degrees of freedom=17 (Chi-square/df= 2.12)		
	Root Mean Square Error of Approximation (RMSEA)= 0.06			Root Mean Square Error of Approximation (RMSEA)= 0.07		
	Goodness of Fit Index (GFI) = 0.98			Goodness of Fit Index (GFI) = 0.96		
	Standardized Root Mean Square Residual (SRMR) = 0.03			Standardized Root Mean Square Residual (SRMR) = 0.05		

**Table 5:** The reliability index and the confirmatory factor analysis (CFA) of colleague-related items

Items back translated from Swedish	colleague group N=203						self-evaluation N=213					
	factors/dimensions factor loadings (t values)					corrected item-total correlation	factors/dimensions factor loadings (t values)					corrected item-total correlation
Make an assessment in the following areas:	knowledge and skills	attitude and approach	teaching	reflection and development	trust		knowledge and skills	attitude and approach	teaching	reflection and development	trust	
Clinical knowledge	0.81 (13.70)					0.73	0.78 (12.94)					0.55
Diagnostic thinking	0.77 (12.73)					0.70	0.75 (12.12)					0.48
Clinical decision making	0.81 (13.52)					0.74	0.74 (11.97)					0.54
Prescribing	0.75 (12.20)					0.71	0.77 (12.56)					0.57
Treatment (Including practical procedures)	0.72 (11.57)					0.67	0.57 (8.82)					0.44
Medical record keeping	0.56 (8.27)					0.53	0.34 (4.71)					0.38
Recognizing and working within own limitations				0.76 (12.40)		0.70				0.52 (6.97)		0.30
Keeping knowledge and skills up to date				0.72 (11.46)		0.69				0.68 (9.29)		0.47
Reviewing and reflecting on own performance				0.76 (12.26)		0.70				0.49 (6.38)		0.43
Teaching (students, trainees, others)			0.86 (9.90)			0.39			0.74 (6.20)			0.41
Supervising colleges			0.67 (8.31)			0.39			0.66 (5.98)			0.36
Commitments to care and wellbeing of patients		0.77 (12.71)				0.70		0.68 (9.85)				0.44
Communication with patients and relatives		0.77 (12.66)				0.70		0.66 (9.50)				0.38
Working effectively with colleagues					0.81 (10.65)	0.49					0.40 (5.57)	0.46
Effective use of time and resources	0.70 (10.93)					0.67	0.48 (6.97)					0.52
Patient-centered approach		0.78 (12.99)				0.74		0.68 (9.81)				0.54
Preserving the continuity of patient relationships		0.74 (11.94)				0.69		0.50 (6.90)				0.48
Respects patient confidentiality					0.42 (5.50)	0.37					0.38 (4.81)	0.13
Trust in this doctor					0.56 (7.52)	0.27					0.81 (13.48)	0.43
Cronbach's alpha						0.93						0.84
	Fit indices CFA for Colleague group (CQ):						Fit indices CFA for self-evaluation, Colleagues part (SCQ):					
	Chi-square = 293.35, degrees of freedom=140 (Chi-square/df=2.10)						Chi-square = 279.89, degrees of freedom=140 (Chi-square/df=2.0)					
	Root Mean Square Error of Approximation (RMSEA)=0.07						Root Mean Square Error of Approximation (RMSEA)= 0.07					
	Goodness of Fit Index (GFI) = 0.88						Goodness of Fit Index (GFI) = 0.88					
	Standardized Root Mean Square Residual (SRMR)=0.06						Standardized Root Mean Square Residual (SRMR)=0.07					

## Discussion

In this study we verified high internal consistency and acceptable construct validity of the Swedish version of the three GMC questionnaires. We determined the underlying components and confirmed two latent variable structure models, one that reflected five aspects of good medical practice for colleague-related items, and another that reflected two latent variables for patient-related items.

The Cronbach’s alpha indices and the PCA in our Swedish version were on the same level as in the UK study.^11^ In both studies one principal component was found in the main items of the PQ and three in the main items of the CQ. Concerning colleague-related items in the Swedish version, five components were identified in the SCQ, while only three were identified in the CQ. 

Self-evaluation data in MSF are sometimes regarded as insignificant and not often analysed in other studies.^27^^,^^28^ However, self-validation has impact in several ways. We noticed during the interviews in the adaptive process, described elsewhere (manuscript in progress), that the residents’ reflections were more complex and different compared to the external assessors when reading the same questions. Underpinned by the residents’ five components in the PCA, we identified an acceptable CFA model with two additional dimensions for colleague-related items in both SC and SCQ. Two added items concerning patient centeredness in the colleague-related part loaded significant in the attitude and approach factor. Together with an emphatic factor in relation to the patient-related items that was verified in the PCA due to a new item about the patient’s concerns in the PQ we found support for a patient-centered approach which is fundamental in our specialist training.

Self-evaluation with MSF in formative assessment is intended to facilitate comparison of scores, and to enable residents to reflect on how their own scores match or miss-match the scores of others who see their work. The UK study explored the correlations and differences between scores of self -assessment by index physicians and those of external appraisers as patients and colleagues and found that the physicians tended to underestimate their own performance.^12^ In our study the residents rated themselves significantly lower in all relevant 5-points items compared to colleagues. Updating skills, to mentor, and to work effectively were items where the residents scored themselves lowest (Table 3) and are interesting signals of possible need for further training, or may also signal a need for the resident to ‘recalibrate’ their own assessments. The SQ scores were more normally distributed than the negative skewed scores in PQ and CQ, and provide better opportunities for analysing progress. One incidental finding in our data was that we noted improvements in clinical decision-making between residents in the first and second part of their residency.  To follow changes in competence of individual residents over time is an interesting task for future studies. To determine the minimum number of respondents in order to obtain reliable scores would also be of interest.

Ceiling effect, the frequency of highest possible score, is a generic problem in MSF^29^^,^^27^ and also in the GMC questionnaires.^11^ A ceiling effect of 15% is regarded as the maximum acceptable cut-off value.^30^^,^^31^ Fewer than 85% of our respondents gave the highest score (Tables 2a, 2b and 3), which was interpreted as acceptable. It is not sure that addition of more response options or scale transformations will solve this problem.

Assessor cognition bias is another problem in MSF described by Gingerich et al.^7^^,^^32^ In the semi-structured interviews performed during the adaption process we found that many nurses and secretaries used the “don’t know” answer options. Some of our co-workers did not think they dared nor had the knowledge to assess a resident. To offer separate answer reports from physicians and other staff could be a possibility to deal with that problem but that requires more assessors. We believe there is a greater educational potential in using the Swedish version as an instrument for formative feedback during specialist training than for summative assessment in a revalidation process as it is used in the UK.

### Limitations

Reliability and validity of the three GMC questionnaires were analysed separately as only one quarter of the residents who performed self-evaluation were assessed by patients, colleagues or both. These circumstances prevented us from performing some statistical analysis, such as correlations between groups and exploration of factors related to index physicians which have been performed in the UK.

A possible limitation in the CQ was the many “don’t know” answers that complicated the calculations. However, most of the “don’t know” answers were due to the fact that residents seldom supervise colleagues and that colleagues seldom take part in residents’ teaching activities.

A further possible weakness is the relatively small sample size of residents who received feedback. The number of external assessors for each resident was also lower than recommended in the UK study.^33^ An explanation for the relatively small sample size might be that MSF is not widely used in Sweden, which can lead to ambivalence among residents towards taking part in MSF. However, the sample size of each questionnaire was more than 200 and the numbers of answers per item were more than 10. Although the number of SQ responses was sufficient, the sample of those residents who received feedback was relatively small and not random. However, the sample did not deviate from the rest of the residents who did not receive feedback. There was also a wide variation in demographic data among participants, which strengthened the results.

There are different opinions about how to analyse results from Likert scales. Some hold that ordinal data ought to be calculated with non-parametric methods as we did in the descriptive and comparative statistics. However, according to Brown, supported by journal editors^34^^,^^35^ parametric statistics, the predominant method, can safely be used even with non-normal distributed Likert data as we did in reliability and validity analyses.

## Conclusions

The study showed that the Swedish version of the GMC questionnaires has high reliability and acceptable construct validity for formative workplace assessments. The CFA validated two acceptable models with the same latent factors for different assessors, which makes comparisons between their assessments relevant. The latent factors are in line with good medical practice. The Swedish version can be used for further testing in larger samples with the aim to assess the clinical competence of residents. The questionnaires could be provided as additional tools for evaluation of progress twice during the residents training. As this is the only validated MSF instrument for physicians in Sweden, it can be beneficial for both resident physicians and their supervisors in family medicine as a feedback instrument.

### Acknowledgements

The authors acknowledge the invaluable contributions of all the patients, colleagues, and residents who participated in this study as well as our project administrator Anneli Lagerqvist for her dedicated administrative help, Helena Schildt-Tossman, MD, head of the vocational training department, Academic Primary Health Centre, for her support throughout the entire project, and Ed Peile, Emeritus Professor in Medical Education at the University of Warwick who gave us the project idea and proofread the manuscript. This research received a grant from The Swedish College of General Practice (SCGP).

### Conflict of Interest

The authors declare that they have no conflict of interest.
